# Autologous Matrix-Induced Chondrogenesis (AMIC) and AMIC Enhanced by Autologous Concentrated Bone Marrow Aspirate (BMAC) Allow for Stable Clinical and Functional Improvements at up to 9 Years Follow-Up: Results from a Randomized Controlled Study

**DOI:** 10.3390/jcm8030392

**Published:** 2019-03-21

**Authors:** Laura de Girolamo, Herbert Schönhuber, Marco Viganò, Corrado Bait, Alessandro Quaglia, Gabriele Thiebat, Piero Volpi

**Affiliations:** 1 IRCCS Istituto Ortopedico Galeazzi, Via Riccardo Galeazzi, 4, 20161 Milano, Italy; herbert@shonhuber.it (H.S.); marco.vigano@grupposandonato.it (M.V.); gthiebat@gmail.com (G.T.); 2 Istituto Clinico Humanitas IRCCS, Via Alessandro Manzoni, 56, 20089 Rozzano, Italy; info@corradobait.com (C.B.); alessandro.quaglia@humanitas.it (A.Q.); piero.volpi@humanitas.it (P.V.)

**Keywords:** autologous matrix-induced chondrogenesis (AMIC), bone marrow aspirate concentrate (BMAC), cartilage repair, chondral lesions, mesenchymal stem cells

## Abstract

The aims of the study were to evaluate long-term outcomes after autologous matrix-induced chondrogenesis (AMIC) in the treatment of focal chondral lesions and to assess the possible improvements given by the combination of this technique with bone marrow aspirate concentrate (BMAC). Twenty-four patients (age range 18–55 years) affected by focal knee chondral lesions were treated with standard AMIC or AMIC enhanced by BMAC (AMIC+). Pain (Visual Analogue Scale (VAS)) and functional scores (Lysholm, International Knee Documentation Committee (IKDC), Tegner, Knee injury and Osteoarthritis Outcome Score (KOOS)) were collected pre-operatively and then at 6, 12, 24, 60, and 100 months after treatment. Magnetic resonance imaging (MRI) evaluation was performed pre-operatively and at 6, 12, and 24 months follow-ups. Patients treated with AMIC+ showed higher Lysholm scores (*p* = 0.015) and lower VAS (*p* = 0.011) in comparison with patients in the standard AMIC group at the 12 months follow-up. Both treatments allowed for functional and pain improvements with respect to pre-operative levels lasting up to 100 months. MRI revealed consistent cartilage repair at 24 months in both groups. This study shows that AMIC and AMIC+ are effective treatments for focal chondral lesions with beneficial effect lasting up to 9 years. AMIC+ allows for faster recovery from injury, and is thus more indicated for patients requiring a prompt return to activity. Level of evidence: II, randomized controlled trial in an explorative cohort.

## 1. Introduction

Articular cartilage injuries represent an issue in current orthopedics surgery, due to the high prevalence in the active population and to the low healing potential of hyaline cartilage [1]. Bone marrow stimulation techniques as well as cell- and tissue grafting-based therapies for the treatment of full thickness cartilage defects have demonstrated satisfactory results [2,3,4,5,6,7,8]. However, these techniques suffer from several limitations. Some approaches, such as autologous chondrocyte implantation (ACI), require two subsequent surgeries for cell harvesting and transplantation with higher costs and risks related to the procedure. Others, such as microfractures of the subchondral bone, may result in unsatisfactory tissue healing, with the production of fibrocartilaginous tissue, and therefore poor long-term clinical results [7,8,9,10]. Autologous matrix-induced chondrogenesis (AMIC) is a matrix-assisted bone marrow stimulation technique combining microfractures with the use of a type I/III porcine collagen matrix (Chondro-Gide, Geistlich Pharma AG, Wolhusen, Switzerland), which is able to stabilize and protect the bone marrow clot deriving from microfractures and containing mesenchymal stem cells (MCSs) [11,12,13]. Given the nature of the matrix and its role as a scaffold, this technique provides a proper stimulus for the chondrogenic differentiation of MSCs while allowing for the treatment of patients in a single surgical stage. Satisfactory results using this approach have been already reported at medium- and long-term follow-ups, with pain reduction and functional improvement up to 7 years [14,15,16,17].

AMIC may benefit from the association with other biological procedures such as the addition of platelet-rich plasma (PRP) that can further stimulate the tissue repair ability of cells deriving from the microfracturing of the subchondral bone [18]. However, since the real amount of MSCs contained in bone marrow from subchondral bone is very low [19], it is possible to hypothesize that the combination of AMIC with bone marrow aspirate concentrate (BMAC) would improve the clinical outcomes given the increased number of progenitor cells located at the lesion site. In addition, this may be crucial in consideration of the lower number of resident MSCs in the joint subchondral bone with respect to the iliac crest [20].

Based on these observations, a prospective randomized study was performed to compare the clinical outcomes of standard AMIC technique versus the AMIC technique enhanced by the use of autologous BMAC harvested from the iliac crest (AMIC+) for the treatment of focal chondral defects. The hypothesis of the study is that the addition of BMAC would have a positive effect in improving the functional outcomes given the higher number of progenitor cells delivered to the injury site. 

## 2. Materials and Methods

### 2.1. Patients

**Figure 1:**
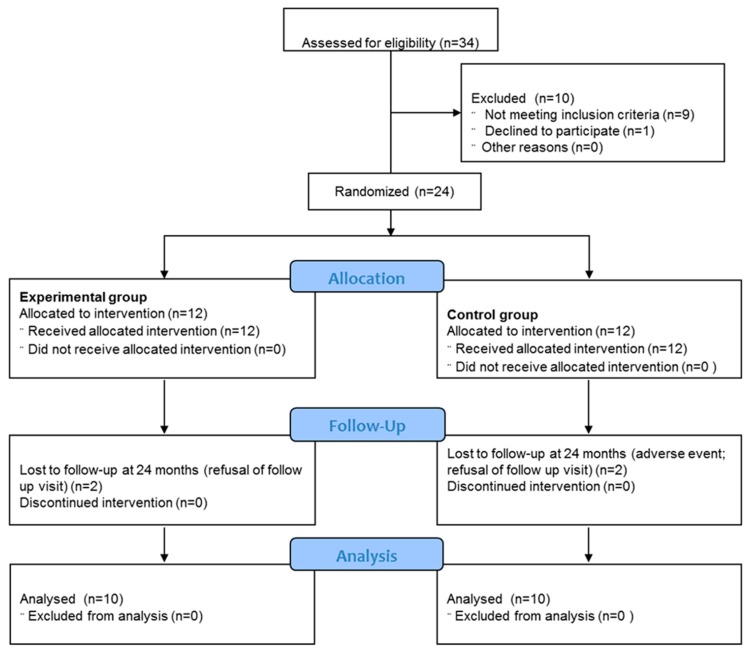
CONSORT 2010 Flow diagram. Patients enrollment, randomization, and dropouts.

The clinical study was conducted after the approval of an external Ethics Committee (ASL Città di Milano, 21/07 MS, protocol n°471/07). Between December 2007 and February 2010, 34 patients with clinical and radiological diagnosis of chondral defects received the indication to undergo an AMIC procedure at our institution (IRCCS Istituto Ortopedico Galeazzi). Twenty-four of them satisfied the following criteria and were included in the study: age between 18 and 55 years, one or two grade III or IV articular surface lesions of the knee according to the International Cartilage Repair Society’s chondral defect classification system (ICRS) [21] (on both tibiofemoral and patellofemoral joint), lesion size between 2 and 8 cm^2^, and normal surrounding cartilage (accepted one or two grade I/II chondral lesions). Patients with more than two chondral defects, immuno-mediated pathologies including osteoarthritis, knee infection, untreatable instability, malalignment or meniscal tears, serious cardiologic pathologies, or problematic general conditions were excluded from the study. All the patients were informed about the study and signed an informed consent. Immediately before surgery, the patients were randomly assigned either to the standard AMIC (group AMIC, *n* = 12) or to the concentrated bone marrow aspirate (BMAC)-enhanced AMIC technique (group AMIC+, *n* = 12) (Figure 1). The randomization process was guaranteed by a randomization list generated by a computer software, blinded in consecutively numbered sealed envelopes assigned to each patient just before starting the surgical procedure. A study co-investigator was responsible for the randomization of the procedure. 

### 2.2. Surgical Technique

The surgical interventions were performed by two senior surgeons. Tourniquet was used for all patients; after checking the inclusion/exclusion criteria during the arthroscopy, the lesion was debrided, removing degenerated cartilage and creating a regular-shaped lesion. For each patient in the AMIC+ group, a sample of 24 mL of bone marrow was harvested from the anterior iliac crest and added to 6 mL of Anticoagulant Citrate Dextrose Solution A (ACD-A). As suggested by several authors, bone marrow was collected by multiple localizations (about 4–5 mL per sampling) to avoid the peripheral blood dilution effect [22,23]; the samples were then centrifuged at 3200 rpm for 15 min (room temperature) at the point of care using the MarrowStim^TM^ device (Biomet, USA, currently named “BioCue”, Zimmer-Biomet, Warsaw, IN, USA) according to the manufacturer’s instructions. At the end of the centrifugation a phase enriched in mononuclear cells, including mesenchymal progenitors, was obtained. Meanwhile, a mini-arthrotomy (2–3 cm, depending on the site of the lesion) was performed to allow access to the defect. Using a template, the collagen type I/III bilayer matrix (Chondro-Gide**^®^**, Geistlich Pharma AG) was then cut according to the dimensions and the size of the defect. Microfracturing of the subchondral bone was performed following Steadman’s technique [24] using specific awls (Chondropic^TM^, Arthrex, Naples, FL, USA). For the standard AMIC group the matrix was positioned on the defect and fixed with synthetic fibrin glue (Tissucol, Baxter, Deerfield, IL, USA). For the AMIC+ group, before being placed on the lesion, the shaped matrix was dipped into the concentrated bone marrow aspirate for 10 min, according to a previously described technique [20], and then glued as reported above. Small amounts of bone marrow samples, both before and after concentration with the MarrowStim device, were collected and the isolated cells were characterized both as fresh cells and after two passages in culture as previously described [20].

### 2.3. Flow Cytometry of Bone Marrow Concentrate-Derived Mesenchymal Stem Cells

The immunophenotype of cells cultured for two passages in human mesenchymal stem cells growth medium (MSCGM–Lonza, Verviers, Belgium) was analyzed by FACSCalibur™ (Becton-Dickinson, Franklin Lakes, NJ, USA) and CellQuest™ analysis software, after staining with the following conjugated antibodies: anti human CD45-PE, CD34-PE, CD31-PE, CD90-PE, CD73-PE, CD166-PE, CD44-PE (all from Becton Dickinson), and CD105-FITC (R&D System, Minneapolis, MN, USA). Cells were incubated with saturating concentrations of the appropriate antibodies for 30 min at 40 °C. 

### 2.4. Clinical Evaluation

The physician in charge of the clinical controls as well as the radiologist were blinded to the allocation of patients in the study groups. Pre-operatively and then at 6, 12, and 24 months from intervention, the Visual Analogue Scale (VAS) [25], Lysholm–Tegner Score [26], objective International Knee Documentation Committee Subjective (IKDC) [27], and Tegner Activity Scale were recorded for each patient. Given the satisfactory results and the lack of data about the long-term outcomes of AMIC procedures, the patients were kept under observation, assessing their conditions at 60 and 100 months. At these longer follow-ups, the patients were interviewed by phone and thus the objective IKDC was replaced by the Knee injury and Osteoarthritis Outcome Score (KOOS) [28].

### 2.5. Magnetic Resonance Imaging (MRI) Protocol

Pre-operatively, all patients underwent magnetic resonance imaging (MRI) with a 1.5 Tesla scanner to assess the location, size, and severity of the chondral defect, as well as any other soft tissue damage including the menisci or ligamentous structures by T1 weighted sequences. Structural regeneration of the cartilage defect was then assessed at 6, 12, and 24 months by an independent radiologist, with a focus on the extent, signal intensity, and surface of the defect filling, integration to adjacent cartilage, and bone marrow lesion (BML). This adapted scoring system takes into account a variety of features that are currently believed to be relevant to the integrity of cartilage repair tissue as used in the Magnetic resonance Observation of CArtilage Repair Tissue (MOCART) score [29] after ACI and semi-quantitative MRI scores of osteoarthritis established as the Whole-Organ Magnetic Resonance Imaging Score (WORMS) [30] and the Boston Leeds Osteoarthritis Knee Score (BLOKS) [31]. 

### 2.6. Rehabilitation Protocol

Both groups followed the same rehabilitation program. In case of condylar chondral defect, immediate full range of motion was allowed without any weight bearing for 3 weeks, then full bearing was allowed after the sixth week; in case of patellar defects, patients were allowed to progressively restore the full range of motion and bearing starting from the early post-operative days. All patients were advised to return to sports after 4–6 months from surgery, depending on the sport.

### 2.7. Statistical Analysis

All data were collected in an electronic database. Analysis of variance was performed on continuous data (VAS Pain, Lysholm, and Tegner scales), whereas the χ^2^ test was used to analyze the categorical score (IKDC). Analyses were performed with Graphpad Prism v5.0 (Graphpad Software, La Jolla, CA, USA). Data are expressed as means ± standard deviations (SD). Values of *p* < 0.05 were considered statistically significant. At the time of project design, it was not possible to perform a reliable sample size calculation due to a complete lack of data about the outcome of AMIC. For this reason, the power of our statistical significances was calculated *ex post* with Gpower software v3.1.9.2 [32]. Differences between different time points in the same group demonstrated 99% statistical power, for both the VAS and Lysholm score, defined as primary outcome measures. The differences between AMIC and AMIC+ at 12 months were characterized by a statistical power of 72% and 60% for the Lysholm score and VAS, respectively. The statistical power of the difference between the IKDC score (secondary outcome measure) in the AMIC+ group (pre-operative vs. 24 months) was 79%.

## 3. Results

### 3.1. Patients

For each group, all the patients (*n* = 12) received the intended treatment they were randomly assigned to and were analyzed for the primary outcome. Two patients for each group were lost within 5 years of follow-up, resulting in 10 patients per group at that time point. In the AMIC group, one of the patients had arthrosynovitis that provoked his exclusion from the study 1 year after surgery. We are not aware of any other failure or patients who underwent a successive surgical procedure. 

Three more patients were not reachable and could not answer the questionnaires in the AMIC group; one patient died due to unrelated causes in the AMIC+ group after the 60 months follow-up. Thus, at the last follow-up the analysis included seven and nine patients in the AMIC and AMIC+ groups, respectively. No acute or long-lasting pain was reported after bone marrow harvesting from iliac crest in any patient. The AMIC+ technique was reproducible and it did not affect the duration of intervention with respect to the standard AMIC procedure, with the exception of the time required to harvest the bone marrow.

### 3.2. Bone Marrow Cells Analysis

In vitro analysis of bone marrow samples revealed a difference in terms of MSC concentration among patients, with an average concentration factor of 3-fold in the concentrated bone marrow samples with respect to whole bone marrow (*p* < 0.05). In all concentrated samples, the nucleated cells demonstrated the typical MSC surface markers expression pattern, as reported in Table 1. 

### 3.3. Demographic Data at Baseline

At the time of enrollment, the background population features (age, weight, lesion size, gender) as well as pre-operative scores were homogeneous between the two groups (Table 2). With the exception of one patient in each group, all the other patients practiced sports, with seven and six of them practicing at a competitive level in the AMIC and AMIC+ groups, respectively. 

### 3.4. Functional and Clinical Outcomes 

**Figure 2:**
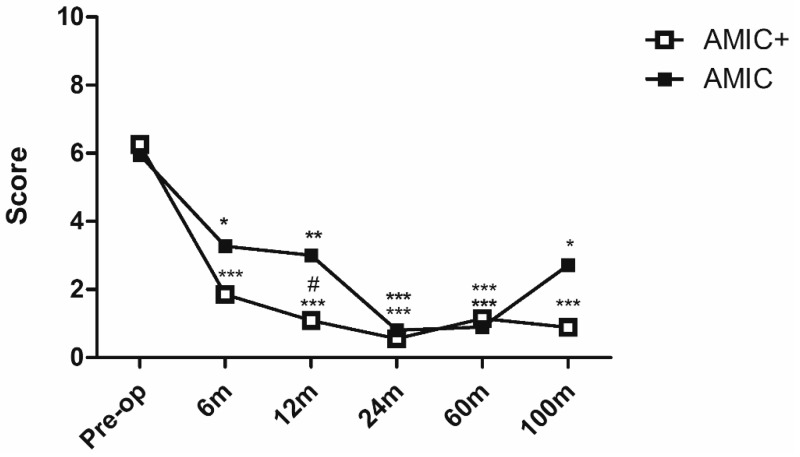
VAS score after AMIC and AMIC+ procedures at up to 9 years follow-up. * *p* < 0.05, ** *p* < 0.01; *** *p* < 0.001 vs. the respective pre-op levels. # *p* < 0.05 vs. standard AMIC.

The VAS (Figure 2) significantly improved 6 months after treatment in both groups (−42% in the AMIC group, *p* < 0.05; −72%, *p* < 0.001 in the AMIC+ group). At the 12-month follow-up, the VAS was not significantly improved with respect to the 6-month assessment, but patients treated with AMIC+ demonstrated a significantly lower pain score with respect to the AMIC group (*p* < 0.05, effect size 1.2, mean difference 1.9, 95% confidence interval 0.5–3.3). This difference was no longer present at the 24-month follow-up, when both groups showed the minimum level of pain recorded (*p* < 0.001 with respect to pre-operative levels in both groups). The VAS remained constant up to 100 months of follow-up, with only a slight increase for the AMIC group at the latest follow-up. However, it was always significantly lower than the pre-operative level (*p* < 0.05). 

**Figure 3:**
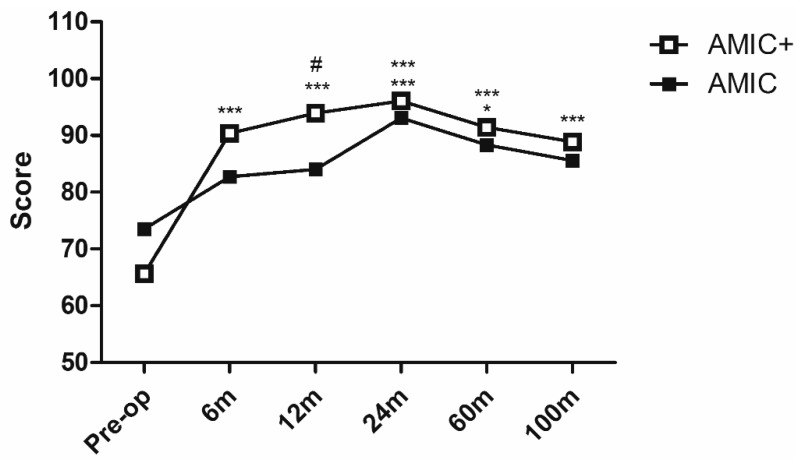
Lysholm score after AMIC and AMIC+ procedures at up to 9 years follow-up. * *p* < 0.05, *** *p* < 0.001 vs. the respective pre-op levels. # *p* < 0.05 vs. standard AMIC.

The Lysholm score (Figure 3) improved in both groups 6 months after surgery, but the difference was significant only in the AMIC+ patients (+14% in AMIC, ns; +39% in AMIC+, *p* < 0.001). A similar situation was observed at the 12-month follow-up, where the difference between the two groups was significant in favor of the AMIC+ group (*p* < 0.05, effect size 1.14, mean difference 9.9, 95% confidence interval 2.1–17.6). After 24 months from surgery both groups presented significant Lysholm score improvements with respect to baseline (*p* < 0.001). At the 60- and 100-month follow-ups a slight progressive reduction of this score was observed, although AMIC+ patient scores always remained significantly higher than baseline (*p* < 0.001 at both follow-ups). 

**Figure 4:**
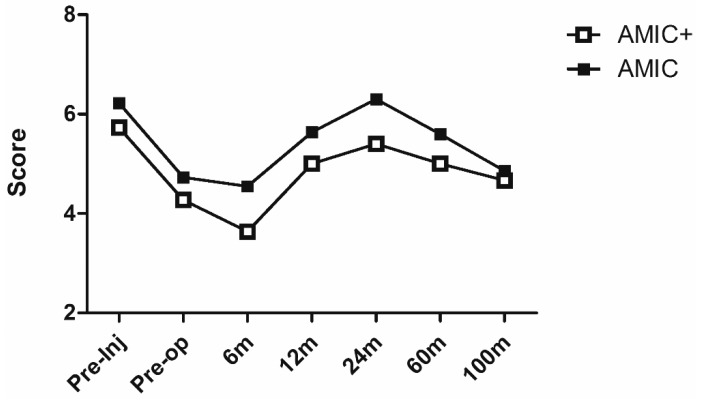
Tegner activity scale after AMIC and AMIC+ procedures at up to 9 years follow-up.

The evolution over time of the Tegner activity scale (Figure 4) demonstrated a return to the pre-injury levels in both groups already starting at the 12-month follow-up, with a further increase up to 24 months. Then a progressive reduction, although not significant, of the sport activity level was observed, which was justifiable as a physiological drop due to the effect of age on sports activity, especially in competitive athletes. Nevertheless, even the scores at the last follow-up were not significantly lower than the pre-injury levels (Figure 4). All the functional scores data are reported in Table 3.

**Figure 5:**
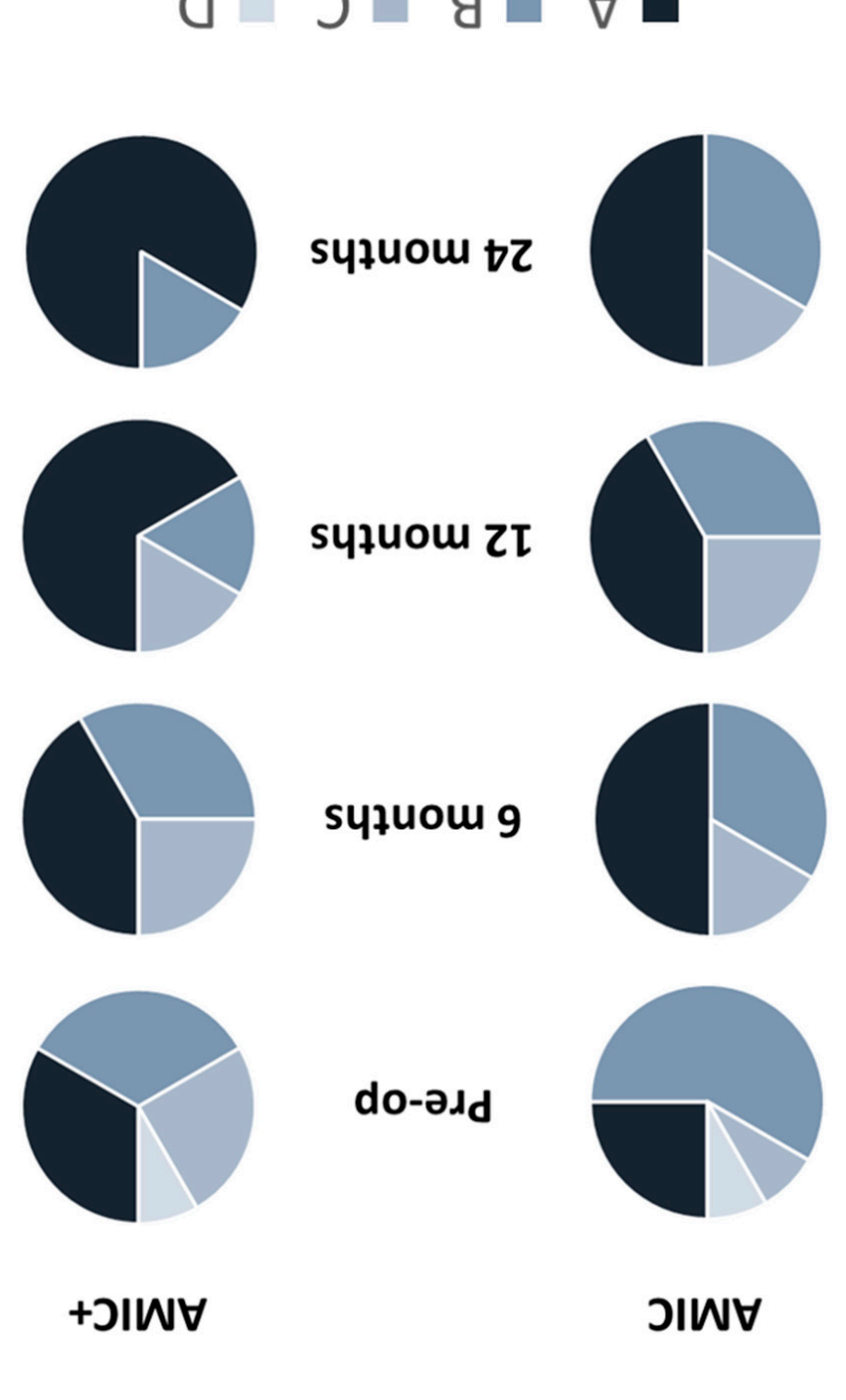
Proportion of IKDC scores in AMIC and AMIC+ groups at different follow-ups. At 24 months after surgery, proportions in the AMIC+ group statistically differed from pre-operative levels, with a higher percentage of patients in category A with respect to the other scores (B,C,D) (*p* < 0.5). No statistically relevant differences were observed in the standard AMIC group.

The objective IKCD scores improved in both groups 6 months after surgery (Figure 5). At later time points, patients treated with the standard AMIC technique did not show further improvements, while the proportion of patients scoring A in the AMIC+ groups improved constantly until the 24-month follow-up. At this time point, the proportion of patients scoring A was significantly increased with respect to the pre-operative assessment (*p* < 0.05). On the contrary, no significant difference was observed in the standard AMIC group at any time point. 

**Figure 6:**
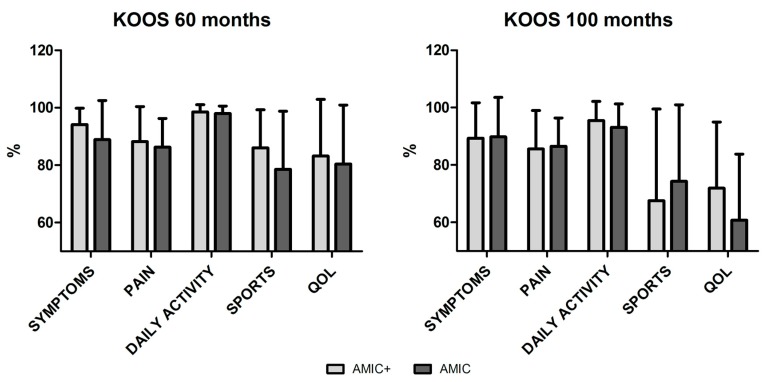
Knee injury and Osteoarthritis Outcome Score (KOOS) in AMIC and AMIC+ groups at 60- and 100-month follow-ups. Patients in both groups showed similar KOOS at both time points. A slight decrease was observed between the 60- and 100-month follow-ups, in particular concerning sports and quality of life (QOL) scores.

In both groups the KOOS score was satisfactory, in particular for pain and daily activities subsections up to the 100-month follow-up. A slight progressive decrease with time was observed for sport and quality of life (QOL). No differences were observed between the AMIC and AMIC+ patients (Figure 6). 

### 3.5. Radiological Observations

**Figure 7:**
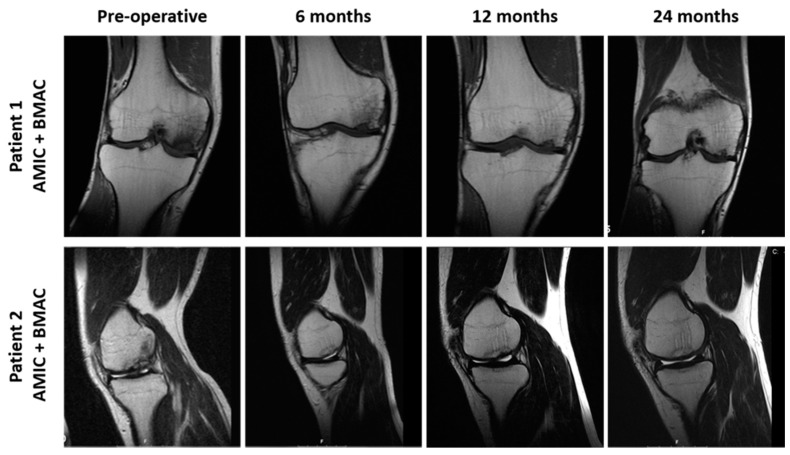
Magnetic resonance imaging (MRI) T1 scans of two patients performed pre-operatively and at 6, 12 and 24 months after AMIC+ treatment.

MRI analysis was affected by a poor patient compliance in the standard AMIC group, where just five and two patients participated at the 12- and 24-month follow-ups, respectively. On the contrary, in the AMIC+ group, just two dropouts were recorded between the 12- and 24-month follow-ups. Nevertheless, it was possible to observe improved surface appearance and MRI signal in the AMIC+ group (Figure 7).

Graft integration was achieved in higher proportions of patients in the AMIC+ group at the 6-month follow-up, while it was comparable between the two groups 12 months after surgery. Defect size and defect filling were similar in the two groups at each follow-up. Reduction in bone marrow lesion was observed in just 30% of the whole cohort of patients (6/20) at 24 months (Table 4); given the very low availability of MRI data in most of the patients belonging to the standard AMIC group at this time point, it is not possible to make any reasonable comparison between groups.

## 4. Discussion

The main finding of this study is that both AMIC and AMIC+ allowed for stable significant clinical and functional improvements in patients with focal knee chondral defects up to approximately 9 years. The patients treated with the AMIC technique augmented with autologous concentrated bone marrow aspirate from iliac crest showed significantly better results in functional recovery with respect to the patients treated by the standard AMIC technique at the 12-month follow-up. MRI analysis confirmed this observation, since a higher proportion of patients showed improvements in the AMIC+ group at the 12-month follow-up with respect to patients treated with the standard AMIC technique, although the decreased number of patients analyzed does not allow for any statistically significant difference.

At later time points the outcome of the two groups were comparable, thus allowing to hypothesize that a larger number of mesenchymal progenitor cells is beneficial in the initial phases of cartilage repair, providing an acceleration of the healing process. 

With the exception of one patient in the AMIC group who developed arthrosynovitis, no other adverse event or complication was recorded. According to the available records, none of the patients required additional interventions to the treated knee during the time of observation, although we cannot exclude this event for the patients for whom we were not able to complete the last follow-up.

To our best knowledge, this is the longest follow-up reported in the literature for the treatment of chondral lesion of the knee with the AMIC technique, as well as the only randomized prospective study providing a direct comparison of the standard technique with that enhanced by BMAC. The very satisfactory clinical results recorded almost 9 years after surgery in both groups of patients showed that AMIC, independently from the addition of bone marrow concentrate, provides stable improvements in patients affected by isolated chondral lesions, even in a population mainly composed by patients practicing high-level sports activities. The AMIC technique consists in the use of a synthetic matrix aimed to retain the blood clot deriving from subchondral bone and to provide a 3D environment on which the progenitor cells can adhere and proliferate, favoring their differentiation process. In fact, it has been demonstrated that bone marrow mesenchymal cells are able to efficiently differentiate in vitro towards different cell types, including chondrocyte-like cells, under proper stimuli such as the 3D culture environment, which enhances the ability of cells to acquire the new phenotype [33,34,35]. However, it is known that the number of progenitor cells contained in the bone marrow is very low, especially in the distal femur or patella [20]. Our results indicate that the addition of mesenchymal stem cells from the iliac crest contribute to a faster functional recovery. This effect could be mediated by both the differentiation ability of MSCs and their paracrine activity that may play an important role in promoting and supporting the regeneration of cartilage tissue by stimulating endogenous cartilage cells. Indeed, nowadays the most accredited rationale of the use of MSCs resides in the possibility to improve tissue healing through their ability to release pro-regenerative mediators able to interact with the resident cells, ultimately leading to the establishment of a more physiological environment [36]. This phenomenon is well described, in particular in terms of the release of anti-apoptotic, mitogen, anti-scarring, and immunomodulatory mediators [1,4,37,38]. Differently from the intra-articular injective use of MSCs, where the only expected role of these cells is related to their paracrine activity, here the use of a cell-seeded matrix positioned on the lesion might justify the idea that MSCs participate, at least partially, in defect healing through their direct differentiation as well. However, a significant difference between the standard AMIC and AMIC+ patients was observed just 1 year after surgery in term of clinical scores. This may lead to the hypothesis that MSCs are responsible for a temporary modulation of the healing process, accelerating the reduction of the local inflammation and resulting in a faster resolution of the symptoms and thus a higher patient satisfaction already at 6 months after surgery. For these reasons, the cartilage repair technique should be associated with BMAC in case of patients with high functional requests such as athletes. Multiple-site bone marrow aspiration aims to reduce the dilution effect by peripheral blood and increase the amount of collected MSCs. This method, together with the use of one of the most recognized systems for bone marrow concentration, allowed for the obtainment of a high quality BMAC.

The stable results observed in this study are consistent with other reports in the literature of long-term follow-ups after AMIC procedures, where Lysholm and IKDC scores remained significantly higher with respect to pre-operative levels [16], as well as the improvement of MRI appearance [17]. However, as mentioned above, it is not possible to compare the results of our patients treated with AMIC+ due to the lack of similar data at long-term follow-ups. 

A paper by Enea et al. [39] reported the retrospective results of nine patients treated with matrix-assisted microfractures (Biocollagen MeRG^®^ collagen membrane, Bioteck S.p.A., Oviglia, Italy) enhanced by BMAC at a 2.5-year follow-up. This study, although suffering from the lack of a control group and a limited follow-up, showed a statistically significant improvement in the mean IKDC subjective score and Lysholm score. Similar to our results, the median Tegner activity scale was not significantly different between the pre-injury and post-operative levels, confirming the efficacy of this technique at least at short-term follow-up. Interestingly, the authors showed the histological analysis of four patients 1 year after the treatment. The biopsies scored a mean overall ICRS II score of 64 and in one case a hyaline-like matrix was found, though it lacked the typical columnar cell arrangement [40]. 

Other biologics were associated with the use of matrix-assisted microfracture techniques, in particular platelet-rich plasma (PRP). In a cohort of 52 patients treated with PRP-augmented polyglycolic-acid (PGA)/hyaluronan scaffold, the authors reported a statistically significant clinical improvement at the 12-month follow-up [41]. Other authors reported the successful clinical association of PRP with AMIC and Chondro-Gide^®^ in a small number of patients; however, these improvements were not confirmed by MRI [18]. 

The main limitations of the present study are the limited number of patients and the heterogeneity of the chondral defect sites. Nevertheless, the statistical power of our observation was above 60% for all the analyses. Moreover, due to the unavailability of objective IKDC scores at the later follow-ups, the long-term outcomes rely on subjective questionnaires, which suffer from high variability among individuals. Another limitation is the poor compliance of standard AMIC patients to the radiological observations at the 24-month follow-up. In addition, a more specific MRI protocol, such as the delayed gadolinium-enhanced MRI of cartilage (dGEMRIC), may have given better and more detailed information about the regeneration process of the cartilage. 

## 5. Conclusions

In conclusion, this study demonstrates that both AMIC and AMIC+ are effective treatments for knee chondral lesions, providing durable results over time. Indeed, the findings demonstrate that both standard and enhanced AMIC procedures assure functional and pain improvements up to 9 years from surgery. AMIC associated with BMAC had better short-term clinical outcome, which could be due to the higher number of progenitor cells in the beginning of the regeneration process, indicating that this augmentation could be considered for patients with higher functional requirements such as competitive athletes.

## Figures and Tables

**Table 1:** Expression of surface markers in mesenchymal stem cells from bone marrow concentrate (BMAC) after two passages in culture.

Marker	Percentage of Positive Cells
CD34	0.8% ± 1.1%
CD45	1.7% ± 1.8%
CD105	98.9% ± 3.2%
CD73	99.5% ± 2.7%
CD166	98.3% ± 2.9%
CD90	99.3% ± 3.1%

Data are expressed as means ± SD.

**Table 2:** Background data of the enrolled patients.

	AMIC	AMIC+	*p*
Age (years)	30.0 ± 10.2	30.0 ± 11.3	ns
Sex	7M/5F	8M/4F	ns
Weight (kg)	69.1 ± 11.5	68.8 ± 12.9	ns
Lesion size (cm^2^)	3.8 ± 1.0	3.4 ± 0.8	ns
VAS pre-op	5.6 ± 2.2	6.3 ± 2.6	ns
Lysholm pre-op	72.2 ± 14.2	64.5 ± 16	ns
Tegner pre-injury	6.2 ± 1.7 (range 3–9)	6.0 ± 1.8 (range 3–9)	ns
IKDC	A + B: 84%; C + D: 16%	A + B: 67%; C + D: 23%	ns
Localization	7 MFC, 3 LFC, 2 PFJ	6 MFC, 2 LFC, 4 PFJ	
Traumatic lesions	2	2	
Previous surgery	6 (DA, TTM, PM, ACLR, PM + DB)	3 (DA, DB, DA + LB)	
Combined procedures	3 (TTM, DB grade II lesion, ACLT)	1 (TTM)	

Abbreviations: VAS = Visual Analogue Scale; IKDC = International Knee Documentation Comitee; MFC = medial femoral condyle; LFC = lateral femoral condyle; PFJ = patello-femoral joint; DA = diagnostic arthroscopy; TTM = tibial tuberosity medialization, PM = partial meniscectomy; ACLR = anterior cruciate ligament reconstruction; DB = debridement; LB = cartilaginous loose body removal; ACLT = anterior cruciate ligament tensioning. For IKDC score: A = normal; B = nearly normal; C = abnormal, D = severely abnormal.

**Table 3:** Functional, pain, and activity scores in AMIC and AMIC+ patients at each follow-up.

	LYSHOLM SCORE			VAS			TEGNER		
	AMIC	AMIC+	*n*	AMIC	AMIC+	*n*	AMIC	AMIC+	*n*
Pre-op	72.3 ± 13.3 (44–89)	65.2 ± 16.0 (33–80)	AMIC 12; AMIC+ 12	5.8 ± 2.2 (2–8)	6.6 ± 2.7 (1–10)	AMIC 12; AMIC+ 12	4.7 ± 2.8 (2–9)	4.3 ± 2.5 (1–9)	AMIC 11; AMIC+ 12
6 months	84.2 ± 10.6 (64–100)	90.4 ± 6.6 (80–100)	AMIC 12; AMIC+ 11	3.3 ± 1.8 (0–7)	1.9 ± 1.4 (0–8)	AMIC 12; AMIC+ 11	4.5 ± 2.0 (3–9)	3.6 ± 0.9 (2–5)	AMIC 11; AMIC+ 11
12 months	84.0 ± 10.6 (65–100)	93.9 ± 6.2 (78–100)	AMIC 11; AMIC+ 11	3.0 ± 1.8 (0–6)	1.1 ± 1.3 (0–3.5)	AMIC 11; AMIC+ 11	5.6 ± 1.9 (2–9)	5.0 ± 1.8 (3–9)	AMIC 11; AMIC+ 11
24 months	93.1 ± 4.3 (90–100)	96.1 ± 3.8 (88–100)	AMIC 10; AMIC+ 10	0.8 ± 0.9 (0–2)	0.6 ± 0.8 (0–2)	AMIC 10; AMIC+ 10	6.3 ± 2.2 (3–10)	5.4 ± 2.0 (2–9)	AMIC 10; AMIC+ 10
60 months	88.3 ± 9.6 (70–100)	91.4 ± 7.2 (76–100)	AMIC 10; AMIC+ 10	0.9 ± 1.4 (0–4)	1.2 ± 1.3 (0–4)	AMIC 10; AMIC+ 10	5.6 ± 1.4 (3–7)	5.0 ± 2.2 (2–9)	AMIC 10; AMIC+ 10
100 months	85.6 ± 9.4 (73–100)	89.1 ± 6.0 (80–100)	AMIC 7; AMIC+ 9	2.7 ± 2.8 (0–8)	0.9 ± 1.1 (0–3)	AMIC 7; AMIC+ 9	4.9 ± 2.5 (1–8)	4.7 ± 1.3 (3–7)	AMIC 7; AMIC+ 9
Pre-injury	-	-	-	-	-	-	6.2 ± 1.7 (3–9)	6.0 ± 1.8 (3–9)	AMIC 9; AMIC+ 12

Data are expressed as means ± SD (range).

**Table 4:** MRI observations in AMIC and AMIC+ patients at different follow-ups.

		AMIC			AMIC+		
		6 Months	12 Months	24 Months	6 Months	12 Months	24 Months
		*n* = 9	*n* = 5	*n* = 2	*n* = 11	*n* = 11	*n* = 9
Defect filling	None	0	0	0	1	0	0
	<1/3	4	1	0	5	2	1
	1/3–2/3	5	3	1	5	3	2
	>2/3	0	1	1	0	4	6
Surface	Largely uneven	6	2	1	2	1	0
	Partially uneven	2	3	1	9	7	4
	Smooth	1	0	0	0	3	5
Signal intensity of defect cover	Hyper	0	1	1	0	0	0
	Iso	1	1	0	5	8	9
	Hypo	6	3	1	6	3	0
Integration	Marginal gap up 50%	3	3	0	0	0	0
	Marginal gap	3	0	1	2	7	5
	Complete	3	2	0	6	4	4
	Not evaluable	0	0	1	3	0	0
Bone marrow lesion	>2 cm	1	0	0	1	1	1
	1–2 cm	1	0	0	3	1	0
	<1 cm	6	4	2	5	9	7
	None	0	0	0	0	0	1
	Not evaluable	1	1	0	0	0	0

The scoring system takes into account a variety of features that are currently believed to be relevant to the integrity of cartilage repair tissue as used in the Magnetic resonance Observation of CArtilage Repair Tissue (MOCART) score [29] after autologous chondrocyte implantation (ACI) and semi-quantitative MRI scores of osteoarthritis established as Whole-Organ Magnetic Resonance Imaging Score (WORMS) [30] and the Boston Leeds Osteoarthritis Knee Score (BLOKS) [31].
